# Embodying Consciousness through Interoception and a Balanced Time Perspective

**DOI:** 10.3390/brainsci13040592

**Published:** 2023-03-31

**Authors:** Olga Klamut, Simon Weissenberger

**Affiliations:** 1 Department of Psychiatry, First Faculty of Medicine, Charles University, 12000 Prague, Czech Republic; 2 Department of Psychology, University of New York in Prague, 12000 Prague, Czech Republic

**Keywords:** self, consciousness, interoception, balanced time perspective, homeostasis, self-regulation, resilience

## Abstract

This review presents current research and scientific knowledge in body mind sciences through the lens of interoception, as a representative of the body; and time perspective, as the representative of the mind. This intertwining dichotomy has been a subject of discourse in many fields, all having the common denominator of consciousness. Our aim is to expand on the congruities of these seemingly deconstructed worlds-of science and philosophy, of the body and the mind, to show that the place of consciousness lies in the zone between these two. Being aware of the body in the present moment. We introduce interoception and time perspective, focusing on how interoceptive signals are depicted in autonomic nervous system (ANS) regulation, and how this relates to the concept of a balanced time perspective (BTP), a highly adaptive psychological characteristic. Time perspective and interoception are also reviewed in the case of clinical conditions. We assess findings on interoceptive pathways in the body, finding convergence with balanced time perspective through the neuroanatomical lens. We conclude with findings that both dysregulated interoceptive states and a time perspective disbalance are recognized as defining features of mental disorders, proposing prospective practical therapeutic approaches, as well as implications for further research in the field.

## 1. Introduction

While it may initially seem nonphysical, consciousness is often considered a biological phenomenon because the state of it is dependent on biological states within the human body. There exists a prevalent discourse of what consciousness is, what it means to increase one’s consciousness, and what the consequences of this are in terms of a human being’s life and agency in the world. To have agency is to be an active participant in one’s life, being conscious of one’s decisions and choices, instead of being unconsciously emotionally and physically reactive, which is considered an automated state of being. In order to reach this point of responding with awareness, we suggest that one must be in the distinct space and time, which increases this susceptibility of greater consciousness.

We base this on the concept that the nature of the human experience is rooted simultaneously in the body and in the mind, both regulated by the autonomic nervous system and both always active in the present moment. A representation of space is the body, and a representation of time is the mind. Interoception defines internal bodily states by understanding the bodies’ subtle signals, regulating not only internal physiology, but also behavioral processes. In the example of the stress response, during which the nervous system is usually highly deregulated, having a developed sense of interoception can act as a mediator between conscious and automatic behaviors. This in turn allows for self-regulation, leading to resilience and post traumatic growth, which is available to everybody—regardless of demographic. The present moment is a prerequisite to interoceptive awareness, which is available by having a balanced time perspective, a calculable notion that we will introduce in this paper. Our aim of this review is to shed light on what it means to embody psychological regulation and homeostasis, resulting in an increase of consciousnesses and agency in one’s life.

The present-day landscape of consciousness research and theories aims at finding models that relate consciousness to the physical domain. The science of consciousness is met with trans-disciplinary connections within the realm of philosophy, psychology, neurobiology, methodology, and evolutionary biology. We would like to briefly contextualize our viewpoint within a few approaches of understanding this profound phenomenon.

The Sphere Model of Consciousness [1] states the dynamics of consciousness to be space, self, and silence. We similarly address this topic through the lens of space and self. The concept of silence is a prerequisite to engage with what is represented from this dynamic [2]. There is also the concept of dual aspect monism [3], which states that the mental and physical are two aspects of one undivided reality that is psychophysically neutral. This nondual and undivided reality is what we consider a co-regulatory relationship between interoception and time perspective. Moreover, addressing the fundamental role of time perception in consciousness is an important missing piece in the consciousness science discourse, as time researchers Kent and Wittmann exemplify in their review on time consciousness [4].

A major scientific concern in consciousness studies and theories is what Seth and Bayne [5] call the measurement problem, which addresses the difficulty of validating consciousness outside of theoretical considerations and an individual’s introspection. A solution would be objective and calculable markers of consciousness, which we propose as time perspective and interoception. Furthermore, the authors of this expansive review on theories of consciousness [5] describe four primary theoretical approaches to consciousness. Our approach fits the category of what they call a higher order theory, where consciousness is seen as a result of the processing of stimuli. In this case, these stimuli would come from the body in the present moment, which is attainable through a balanced time perspective. We consider this a bi-directional, co-regulatory relationship between the two. The call for scientific formalization and a coherent and consistent framework are the foundations for consciousness theories [6], which we aim to address in our approach.

For additional clarity, we would like to differentiate between two terms used in this paper—time perspective and time perception. Time perspective is a theoretical construct of a cognitive orientation towards the past, present, and future. The concept of a balanced time perspective, a measurable psychological characteristic, is derived from this source. Time perception is the subjective estimation of the passing of time, and can be seen as bound to, and influenced by both time perspective, and interoception.

## 2. The Body—Interoception

William James stated in 1884 that “perhaps philosophers should study physiology, and physiologists should study philosophy” [7]. The study of mind–body interactions had profound advancements due to neurological developments, giving rise to the term of interoceptive awareness. A definition that has been brought forward is “the body’s ability to receive sense information from receptors that respond to internal body states and then use that information to help regulate all of the functional systems of the body” [8].

The body mind connection is vividly described by Stephen Porges in polyvagal theory, which posits and explains the autonomic nervous systems role in maintaining homeostasis [9]. The theory divides the ANS into three subdivisions (the ventral vagal, dorsal vagal, and sympathetic nervous system), each with its own somatic and behavioral responses, depending on the amount of stimulation to the nervous system. Polyvagal theory serves as a tool for placing an individual on the spectrum of sympathetic and parasympathetic activity through the lens of interoceptive signals that show up in various degrees of ANS arousal. A regulated autonomic nervous system contributes to the bodily homeostatic state of balanced mental and physical health. A dysregulated ANS is associated with disbalances in various bodily systems, such as the hormones or digestion, resulting in inflammation and chronic pain. This domino effect of a dysregulated autonomic nervous system influences the entire body. We use this knowledge to conceptualize the autonomic nervous system as a direct bridge between psychology and physiology.

What are often described as ‘gut feelings’ are interoceptive sensations of a homeostatic function taking place in the body. The gastrointestinal tract responds to emotions and stress, as there is a continuous flow of information between the brain and the digestive system [10]. These gut sensations allow individuals to consciously autoregulate, both emotionally, as there is a reciprocal relationship between interoceptive information and emotions [11], as well as physically, such as a response to take a few deeper breaths when a stressful situation causes shallow breathing and an increased heart rate. These biomarkers are associated with the flight or fight response of the polyvagal theory [9].

Furthermore, the entire process of maintaining bodily homeostasis uses interoceptive processes, which include conscious and unconscious levels of cognitive processing, coming from exogenous and endogenous sensory information. These processes include, but are not limited to, physical cues such as heartbeat, breath, thirst, hunger, desire, and pain [12]. Interoceptive signals inform the individual about the homeostatic state of their body, regarding internal (such as cardiac functions) and external (such as touch or smell) stimuli [13]. By being able to explore these signals as they happen, a concept known as interoceptive sensitivity [14], individuals can come closer to a homeostatic state with greater awareness and self-efficacy.

In recent years there has been a rise in interest in the topic of interoceptive awareness due to findings highlighting its integral role in emotional experience, self-regulation, decision-making, and consciousness. In an analysis of the concept, Nayla Khoury and team [12] distinguished between a broad sense of the term, which is the way an individual relates to, processes, integrates and regulates what they are sensing, as well as a narrower understanding of how accurate that sensory process actually is. This has been extensively studied and expanded to include autonomic, hormonal, visceral, and immunological functions such as breathing, blood pressure, cardiac signals, temperature, digestion, thirst and hunger, sexual arousal, touch, pleasure, and pain [15]. The analysis of Khoury et al. [12] consisted of 14 randomized controlled trials with interoception-based interventions and found 50% of them to have a diminishing impact on symptoms of various psychiatric disorders, including anxiety disorders, eating disorders, psychosomatic disorders, and addictive disorders. Their study constitutes a part of a growing body of scientific research linking physiological states to emotional responses.

Recent advancements in neurology have found the exact interoceptive pathways in the body, which take somatic and visceral signals from sensory receptors, through the spinal cord and brainstem, up to the higher cerebral cortical area. Many brain structures, at the forefront being the insular cortex, take part in interoceptive processes, overlapping those responsible for conscious feelings, subjective awareness, and information processing [12]. In terms of sensory physiology, interoception comprises input from internal mechanoreceptors, chemoreceptors, and the afferent sensory part of the vagus nerve [16]. Distinguishing tools to measure interoception is an ongoing debate and there are various methods ranging from qualitative self-reports to quantitative biomarker measurements.

## 3. Measuring Interoception

Measuring interoception is most often done quantitatively, and the most frequent measure used in research is that of heart rate variability (from now on referred to as HRV). HRV is the constant variation between heartbeats, which is counted in milliseconds. While heartbeat is the number of beats per minute, HRV is the space between those beats, which can be more (high frequency HRV) or less (low frequency HRV) constant and linear. The intervals differ in various settings and situations, which reflect the heart’s ability to respond. A higher HRV has been found to be associated with reduced morbidity and mortality [17], improved psychological well-being, and quality of life [18].

HRV is regulated by the autonomic nervous system (ANS) and its sympathetic and parasympathetic branches and is commonly used as a biomarker of the ANS activity [19]. The sympathetic branch activates stress hormones, increases the cardiac output (faster heart rate), and decreases HRV. This reaction is associated with stress. The parasympathetic branch is characterized by a slower heart rate and an increase in HRV, which in turn restores homeostasis, therefore considered the calming response of the ANS [20]. Therefore, HRV can serve as an index of self-regulatory strength [18]. While this is a common and widely accepted measure, it is important to state that associating the ANS branches with higher and lower frequency HRV is prone to criticism as it is considered to be too much of a simplification [21].

HRV is considered the most powerful and subtle signal of the body, as it reacts before the resting heart rate [22]. What was once measured by the polygraph, a device used by the first psychophysiologists to transform unobservable psychological processes into measurable physiological data, is now readily available not only in the lab—but through personal biofeedback devices [23]. Measuring not only the popular somatic measure of HRV, interoceptive trackers such as the Apple Watch, Whoop, or Fitbit bracelets, give access to long term health behaviors and assess body signals, in order to gain control over the most efficient ways to regenerate and consciously come into a homeostatic balance- body and mind. A popular word in the world of fitness and productivity, biohacking is a term coined for the process of monitoring the state in which our bodies are in at a given moment, in order to reach a goal within optimal performance. For example, since one of the ways to increase HRV is deep breathing, one can implement breathing exercises when noticing that the measure is at a lower level. Besides respiration, HRV is also acutely influenced by stress and recovery, hormonal reactions, metabolic processes, cognitive processes, as well as exercise [18]. This physiological indicator of autonomic nervous system activity can also inform on emotion regulation [24]. Recognizing biomarkers that indicate psychophysiological stress is an advance in the field of preventive health and doing so can help individuals understand how their behaviors affect their nervous systems and bodily functions. A consequence of this is the understanding of how to respond to stress in a proactive, healthier way.

Apart from biofeedback devices, interoception is often measured with the heartbeat perception task, during which participants are asked to count their own heartbeats or judge the synchronicity of their heartbeats with an external stimulus [25]. The measure obtained by the heartbeat perception task is interoceptive accuracy, which correlates positively with self-awareness and measures of empathy, prosocial behavior, efficiency in making decisions and emotional sensitivity [26,27]. In order to have a clear overview of the measure, it is important to state that the heartbeat perception task has its share of criticism [28], e.g., that it is influenced by non-interoceptive processes [29], such as individual beliefs about heart rate [30]. Solutions to these limitations are strived for in research, such as within the biochemical determinants of interoception accuracy [31].

Interoception can also be determined using standardized self-report questionnaires; the most frequently used being the Multidimensional Assessment of Interoceptive Awareness [32], with questions such as:

*‘I notice changes in my breathing, such as whether it slows down or speeds up’*

*‘When I am in conversation with someone, I can pay attention to my posture.’*

Apart from this questionnaire, there are a few other condition-specific questionnaires. Examples are the Eating Disorder Inventory EDI [33], with an entire scale of interoceptive deficits, and the Body Perception Questionnaire, developed by Dr Stephen Porges of the abovementioned polyvagal theory [34]. Another worth mentioning is the Scale of Body Connection, one of the first bodily awareness measures and one that includes a scale specific to dissociation from the body [35], therefore the negation of interoception.

There is also a body of growing research on the topic of interoception and mindfulness. Common tools used in this area include the Mindful Interoception Sampling Task (MIST), which defines the concept of mindful interoception as ‘a sustained, non-evaluative present moment attention to endogenous physical sensations occurring internally or at the surface of the body’ [36], as well as the Five Facets Mindfulness Questionnaire (FFMQ), which contains the subscale of ‘Observing’, which includes bodily sensations [37]. Both tools were the key research components in a study conducted by Hanley and team, which examined multivariate networks of association (these being: Regulatory Awareness and Acceptance in Action) between interoception and mindfulness, with scores on a measure of psychological well-being. They found that the two are tightly interwoven, laying a foundation towards future research on how being in the present moment is a vital part towards feeling one’s own body [38]. The aspect of mindfulness itself is inherently grounded in time perspective, and its role on wellbeing is a growing interest in current research [39].

## 4. The Mind—Temporality and Time Perspective

This brings us to the concept of time, a foundational element towards being able to distinguish interoceptive signals and develop sensitivity towards them. Similar as to how one would not know that one has a body without the cognitive understanding of it, we also conclude that one cannot sense their body’s signals without being oriented in the present moment. The aspect of being in the present moment is key to addressing the concept of interoception. One cannot sense bodily signals other than those happening in the distinct moment of cognitively noticing them, such as, i.e., the breath. This aspect is also supported by the shared mechanisms of interoception and time perception [40], which will be assessed in the next sections. A conceptualization of the various aspects of time perspectives, proposed as the Time Perspective Theory [41] results in differentiating between five temporal categories: the past negative, past positive, present hedonistic, present fatalistic, and future orientations. An operationalized tool, the Zimbardo Time Perspective Inventory (ZTPI), is commonly used to place an individual on a spectrum of the five time dimensions, revealing one’s time perspective (TP) profile. Each TP profile is a range of values, emotional attitudes, and various ways to respond with specific behaviors to a variety of life’s situations. The time perspectives transform in the psyche into mental paradigms, a way of interpreting reality [42].

The following Table 1 shows the general characteristics of the five Time Perspectives [43]:

The five ZTPI scales can be read as an extension of the individual relation between an individual and time. Such a relation is defined as a subconscious process, in which an individual places their personal and social experiences within five temporal categories. It is important to state that the ZTPI creates an individual time perspective profile, where an individual is placed on a spectrum of each of the five time perspective profiles. Researchers presented convincing evidence that the use of such an individualized approach to the past, present, and future leads to measurable developmental results in fields such as academic performance [44], risky behaviors [45], stimulant use [46], the frequency of physical activity [47], as well as cognitive functioning [48]. Many studies have been conducted to identify the characteristics attributed for the various TP profiles. A past positive time perspective highly correlates with high self-esteem, a sense of safety, as well as higher levels of amiability and general life energy [49]. This time perspective is also common amongst individuals who tend to be more emotionally intelligent [50]. On the opposite side of the spectrum, the past negative has been found to correlate with a tendency towards depression, addiction, low self-esteem, and problems with forming social relations [51]. A predominance of the present time perspective is present within a tendency towards risky behaviors, such as speeding, excessive drinking, or drug use [52]. In turn, the future time perspective profile is mainly associated with a high level of internal control and a general positive affect [53].

## 5. Balanced Time Perspective

The research around the time perspective profiles shows that often a certain time perspective is prioritized and emphasized in behavior and way of being, which is called a time bias towards the past, present, or future [45]. A temporal profile that is not balanced between the five perspectives, broadly indicating mental health, leads to an excess or deficiency in some areas. This narrowing of the temporal perspective has its consequence in inadequate ways of coping and unhealthy actions in everyday life, which become a relatively constant predisposition. Such a narrowing is not healthy for the human psyche and often has to do with a series of negative consequences, which are usually associated with negative stress coping strategies and unfavorable life conditions [54]. In search of an answer to the question of which time perspective is healthiest, Zimbardo and Boyd [45] proposed an integrated temporal scheme—the balanced time perspective (BTP). According to them, a balanced time perspective is a combination of high scores in past positive, moderate scores in future and present hedonist, alongside low scores in past negative and present fatalistic.

In order to develop a balanced time perspective, to be flexible and have the ability to adapt to a current situation, one has to first obtain an awareness of their own time perspective profile, therefore where they are on the spectrum of each of the five time perspectives [55]. In other words, one needs to know which time perspective profile they have too much or too little of, in order to adapt practices and approaches towards balancing these profiles out to reach the desired balance. This is an analogous process to that of the body coming back into its natural homeostatic state, as in the polyvagal theory. Moreover, successfully shifting between time dimensions results in profiting from past experiences and being aware of future responsibilities [56]. Czesław Nosal, a Polish psychologist from Wrocław who has focused his scientific research on the aspect of time, accentuates that one can:

*“Separately consider and analyze the regulatory significance of the past, present, and future; but the basis of the existential experience of time lies within a mentally recognized totality”*

[57]

We attribute this mentally recognized totality to the concept of the balanced time perspective (BTP), which is an integration of time perspective specific personality traits and behaviors that correspond to mental and physical health, as well as proper social functioning. Simply put, a balanced time perspective profile is an adequate representation of mental health. BTP is measured as high results in past positive and future (the functional orientations), moderate results in present hedonistic, and low results in past negative and present fatalistic (the dysfunctional orientations) [58,59]. Another way of measuring BTP is the Deviation from a Balanced Time Perspective (DBTP) coefficient [60]. This measures the distance of an individual from an optimal TP profile (BTP). Therefore, the higher the distance of DBTP from zero, the more disbalanced an individual’s TP profile is. An additional measure was developed three years later, measuring the Deviation from a Negative Time Perspective (NBTP), as an alternative to measuring the deviation from a maladaptive TP profile [61].

Behaviors and traits that are representative of a balanced time perspective, include- a sense of ambition towards future goals, optimism, reasonable diligence, a natural tendency to see the consequences of one’s behavior, emotional intelligence, a high level of inner control, as well as believing in one’s own self-sufficiency, and a reasonable self-esteem [62]. Further characteristics include a low sense of fear, a low inclination towards depressive states, and a lack of problems with stress coping. It is quite reasonable that present fatalist and past negative time perspectives are not beneficial for one’s health. Research shows that a high bias of these time perspectives is significantly related to low levels of self-realization and lower positive expectations [63], as well as a proneness to PTSD symptoms [64]. Significant correlations between BTP were found in an interaction with life satisfaction [65], a sense of happiness and mindfulness [66], as well as a higher level of emotional intelligence [50]. There have also been preliminary studies [67,68] correlating time perspective with biological markers, such as stress cortisol levels. In one study by Olivera-Figueroa et al. [69], results have shown that the greater the deviation from the BTP, the greater the cortisol systemic output. Stress levels are highly associated with burnout syndrome, also found to relate to a balanced time perspective [70,71]. This points to a direction in which TP profiles are associated with stress physiology, which calls for the exploration of a psychobiological modulation of time perspective and bodily signals, as measured by the interoceptive awareness of an individual.

## 6. Time Perception in Clinical Conditions

Being able to decipher between the past, present, and future is key to being a functioning individual in today’s world. The estimation of time duration is considered a fundamental basis for cognitive and behavioral processes. Pathophysiological distortions in time perception (in the seconds-to-minutes range) are presented by several clinical conditions [72]. The term ‘clinical condition’ is used for describing medical diagnosis falling onto the psychiatric (i.e., schizophrenia, depression, eating disorders) and neurological (i.e., ADHD, Parkinson’s disease, and autism) spectrum.

In an extensive meta–analysis by Vicario and team [73], the above conditions were examined in terms of timing deficits and their relation to insular activation (therefore the neurological home of interoceptive signaling). Their review concluded that there is a strong link between alterations in time processing and dysfunctions of the interoceptive system, suggested by abnormal activity of the insular cortex. This could lead to promising future implications of working on the interoceptive awareness of individuals with clinically diagnosed conditions. It is important to note that insular abnormalities manifest differently in various clinical disorders, which calls for further research in how interoceptive awareness differs within specific conditions [74].

A similar conclusion was drawn by Davalos and Opper [75], who focused solely on schizophrenic patients and their time processing abilities. The disruption of time continuity and perception is a key aspect of the illness. The insular cortex was pointed out as a key region with reduced function in this population, which seems like a direct contribution to timing deficits. The authors made it clear that temporal dysfunctions are part of the psychopathology of schizophrenia and that concentrating more on the time processing dysfunction in the illness, could be key to improving cognition and functioning in this population [75]. Moreover, schizophrenic patients also experience grave alterations to the sensitivity of their internal body signals, again pointing towards the role of insular dysfunction in the disturbance of both interoception and time perception [74].

If interoception and time perception are in such an intricate symbiosis, as our hypothesis points to and many emerging studies confirm, the question persists whether working with the interoceptive system could be a way to regulate time deficits and overall mental wellbeing—especially within clinical conditions. A direct contribution to this hypothesis will be found in adopting time processing protocols more directly related to interoceptive functions, as advised by Vicario and team [73] in their review. Adding measures of the simple biomarkers that we previously mentioned in this review provides additional results as to where an individual is on the spectrum of interoceptive awareness and time perception, highlighting their mental state and the knowledge on how to adjust it. This raises numerous possibilities for interventions, such as somatic therapies for psychiatric patients.

## 7. Congruence—Interoception and Time Perspective

Interoception and balanced time perspective (BTP) are both innate concepts that the human cannot function without—interoception predominantly representing the body, and BTP predominantly representing the mind. For clarity, we use two distinct terms relating to time. Time perspective refers directly to Zimbardo’s time perspective theory and its constructs, whereas time perception is a broader term referring the subjective experience of time and how an individual interprets the duration of an event [76].

A global connection between time and interoception is the fact that both are inherent parts of the present moment. The perception of time is essential for the survival of the individual organism [77]. The body constantly regulating and working towards homeostasis is the foundation of human survival. For further interest, this concept is interestingly explained in the example of the gut–brain connection [78]. There is a wide array of research focusing on either interoception or time perspective in regard to their effect on mental and physical wellbeing, both coming to overlapping behavioral findings. A thorough review done by Farb and team [79], focusing on the role of interoception on overall health in terms of contemplative practices, distinguishes various positive consequences to a greater sense of interoception, the following which are congruent to those of a balanced time perspective, as mentioned in the previous section: greater emotional regulation, an increased perception of presence and agency, increased cognitive flexibility, and increased positive experiences [80].

To further emphasize the potential relationship between balanced time perspective and interoception, two recent articles focusing on the connection of these two concepts were reviewed. The first, by Teghil and team [80], focuses on the effect of interoception on the accuracy of time duration perception. Participants were asked to reproduce regularly and irregularly spaced audio cues. The results showed that individual interoceptive awareness (qualitatively signified using the Self-Awareness Questionnaire) predicted a more accurate duration perception in the case of irregular tones. This study points towards the direction that interoception may be more likely engaged when there is no pattern or regularity in cues to discern from. The duration reproduction task was also the key focus in the second study that we reviewed, alongside a heartbeat perception task. The methodology here was far more quantitative, as skin conductance (an indirect measure of sympathetic autonomic activity) and cardiac and respiratory periods were recorded. Researchers Meissner and Wittmann [81] concluded that participants with a greater interoceptive awareness were able to reproduce time intervals more accurately.

A clear connection is also confirmed through the neuroanatomical lens, as the insular cortex serves as the underlying neural system for both interoceptive and time perceptual functions. Thoroughly identified by neuroanatomist Bud Craig, the insula has demonstrated to be significant for the evolution of human awareness of feelings from the body [82]. He proposed that the posterior insular cortex contains a sensory representation of interoceptive activity from sympathetic and parasympathetic nerves, relating to the physiological condition of the entire body [16]. His findings gave rise to the model of homeostatic emotion regulation [83]. This model proves that the sense of time passing slowly is associated with the right forebrain and broad negative affect, which in turn is a representative trait of sympathetic nervous system activation. In comparison, time passing quickly is associated with the activation of the left forebrain, bringing about feelings of positive affect and being associated with parasympathetic activity. Wittmann [84] and Teghil and team [80] also confirmed the insular cortex as a source of time perception in the brain in their independent studies focusing on fMRI brain scans.

In their extensive research on this topic, Pollatos and team [85] found that retrospective temporal distortions (how and what we remember in time) are directly influenced by attention to bodily responses. Their research led to the conclusion that temporal effects might crucially interact with arousal level and that sympathetic nervous system activation affects memory build-up, which might be the decisive factor influencing retrospective time judgments. Similar findings were described in an extensive review of time perception mechanisms in the central nervous system, acquired by Fontes and team [76]. This conceptualizes that the same neural representations are responsible for the awareness of time and the physiological conditions of the body [85]. Positive correlations between HRV, a measure of interoception, and the accuracy of reproducing the same time durations, were also found [81].

Craig’s theory is closely connected to the aforementioned polyvagal theory of Stephen Porges, explaining the homeostatic function of the autonomic nervous system. An in-depth review of 134 publications reporting emotional effects on peripheral physiological responses, conducted by Kreibig and team [19], distinguished patterns within the ANS, which characterize specific emotional states, giving further information on the behavioral determinants of the state of the autonomic nervous system. This evidence constitutes Craig’s theory of homeostatic emotional regulation. Pollatos and team [85] took this even further in their study, focusing on the emotional qualities of fear and amusement, two seemingly opposing emotional states. They concluded that the emotional qualities of fear were associated with subjective time dilation (time perceived as seemingly longer), and subjective time contraction for amusement (time perceived as faster). Their research brought about the hypothesis that paying attention to interoceptive signals induces a more intense perception of bodily changes, which emphasized the effect of emotional states on subjective time experiences [85].

In the Table 2 below, we present findings from the studies mentioned in this section, visualizing the spectrum of functioning based on time and interoception in the left and right forebrain:

The left side of the table shows approachable behavior and physiological restoration, while the right side identifies that of a physiological response to fear. This serves as preliminary evidence that subjective time, a psychological concept, is the physiological representative of nervous system activation [76].

An increasing number of studies are focusing on the biomarkers of stress and relating them to time perspectives. Ref. [86] concluded that acute stress is modulated by the time perspectives, focusing on neuroendocrine, immune, metabolic, and cardiovascular biomarkers. Chronic sympathetic nervous system activation, therefore being stuck in a state of physiological stress, has many pathological consequences in the form of diseases [87]. Deregulated interoceptive states are becoming increasingly recognized as a defining feature of mental illness [15], such as a disbalanced time perspective (measured by a deviation from the balanced time perspective), which is correlated with depression, anxiety, and stress [88].

## 8. Conclusions

The simultaneous goal and method of increasing both interoception and balancing an individuals’ time perspective is coming into the present moment. This allows for a greater susceptibility to building interoception—one must focus on what is happening in the body in order to be receptive to it. Having a developed sense of interoception is a mindful and conscious awareness in the present moment, therefore bringing an individuals’ time perspective into a greater balanced state. The psychometric tools used to define the two concepts allow for an exact score of the individual on a spectrum of progress. This spectrum being a lesser/greater sense of interoception and a less/more balanced time perspective. This information acts as a starting point in treatment.

There are many practices aimed at developing interoceptive awareness and balancing out an individuals’ time perception. The practice of yoga has shown to influence not only interoception [79,89,90] but also time perception, as studies have shown shorter reaction times and increased accuracy after yoga practice [91]. The research on the neuroprotective effects of yoga, meditation [92], and mindfulness practices [93,94,95] is quickly rising in popularity and becoming an important aspect of mental health. Other bottom-up practices (body influencing mind), such as somatic experiencing [96], are often being used in trauma therapies and interventions. A top-down approach (mind influencing body), focusing on the cognitive experience of time, is Time Perspective Therapy (TPT), an extension of cognitive behavioral therapy, which is based on the argument that working with an individuals’ time perspective is a vital component of treatment. TPT has demonstrated to be effective in alleviating PTSD and depressive symptoms [54,97,98]. Moreover, using interoceptive increasing practices in times of crisis could potentially be a great stress reliever, as collective trauma emergencies, such as the recent COVID-19 pandemic, have proven to alter how individuals perceive time [99].

Our hypothesized relationship between interoception and a balanced time perspective requires experimental proof, which will further explore their impact on each other and upon the regulation of the human nervous system. We believe the commonalities between the two serve as a valid framework on which to base further research endeavors. Interesting results could be obtained from future research on the impact of bottom-up, somatic practices on a balanced time perspective, as well as top-down therapies such as TPT, on interoception. We strongly recommend that further research includes critical evaluations of body/time awareness to ensure a standardized use of these concepts. Moreover, longitudinal data on the bi-directional impact of the two analyzed constructs would be a valid addition to the knowledge within body mind sciences. Here we would like to highlight that in the conceptualization of bottom-up and top-down processing, the significant difference is the starting point of information, whether the initial stimuli is coming from the mind (i.e., talk therapies) or the body (i.e., somatic therapies). After this first catalyst, the process is a two-way street of interdependency between the body and mind.

The field of time perspective is also rapidly developing, as researchers dive deeper into Zimbardo’s theory and broadening the TP profile towards new time perspectives. Such new constructs are the future negative time perspective [100], the present eudaimonic time perspective [101], and the expanded present time perspective [102]. The last two, being extended versions of the present time perspective, are particularly related to a balanced time perspective, mindfulness, and feeling the signals of the body. Defining the optimal values of all three of these new time dimensions could also be an interesting prospective research topic, as doing so can incorporate them into new operationalized concepts of the balanced time perspective. There is a lot of work to do within this field. While we focus on the balanced time perspective as a construct, there is also no research regarding how the optimal values of all other individual TP profiles are connected to sensing the body. In terms of a connection to interoceptive awareness, this could be especially interesting regarding all four of the identified present time profiles (hedonistic, fatalistic, eudaimonic, and expanded).

With growing interest in interoception and time perception in cognitive neuroscience and related fields, there is more information available regarding interoception [103,104] as it is a more feasible construct to obtain results from [105]. The neural mechanisms of time perception are not yet fully understood, therefore there are still many questions left within this concept, and consequently that of balanced time perspective. This is a gap in research with very promising results. One of these gaps is how balanced time perspective is associated with mindfulness, as this scope could serve as important evidence towards the efficacy of mindful meditation practices, a vastly researched and practiced field. A goal of such research would be to explain the co-regulation of interoception and BTP, whilst providing tools and practices for the flexible self-regulation of the nervous system and facilitating resilience.

In this review, we present the concept of embodying consciousness through non dualizing between both time and space and mind and body. We aimed to show the convergences between the two, on the example of balanced time perspective, and interoception. Our intention was to expand the notion of the readers, showing that these are two sides of the same coin, resulting in an expanded self-awareness when increased. We hope to spark further interest and demand in research to expand this discourse, which has so far primarily focused on the mind. While valuable literature has been written on this subject of embodiment and consciousness [106,107,108], we believe that the future direction of consciousness science lies within the body and its bi-directional relationship with the mind.

## Figures and Tables

**Table 1:** The five temporal perspective and their characteristics, according to Zimbardo Time Perspective Theory.

Time Perspective	Characteristic
Past positive	Going back to positive memories from the past, attempting to recreate them A sense of security Nostalgia Excess: lack of development in the present
Past negative	Pessimistic approach to past events Reconstructing past negative events or trauma’s, inability to let them be Interpreting present situations through past experiences Emotions associated with traumas, often regret and pain Excess: tendency to freeze in development due to a fear of repeating the past
Present hedonistic	Living here and now, strictly associated with pleasure Intensive way of life Avoiding routine and boredom Tendency to take risks Excess: lack of taking consequences for actions, not taking care of health
Present fatalist	Tendency to undermine the present as ineffective towards future goals Belief that one does not have an influence on his/her life Low self-esteem, low external control Stagnation, helplessness Excess: pessimistic, passive way of perceiving life
Future	Awareness of goals and benefits associated with them Success and prize oriented Setting realistic goals Internal control, proactive way of life Excess: lack of spontaneity, excessive meticulousness, not living in the moment

**Table 2:** Comparative findings.

Left Forebrain	Right Forebrain
Time passing quickly: subjective time contraction	Time passing slowly: subjective time dilation
Positive affect	Negative affect
Parasympathetic nervous system activation	Sympathetic nervous system activation
Greater interoceptive sensitivity	Smaller interoceptive sensitivity
Increased HRV	Decreased HRV
Decreased electrodermal activity	Increased electrodermal activity
Physiological restoration	Physiological stress

## Data Availability

No new data were created or analyzed in this study.
